# Letermovir for cytomegalovirus prophylaxis in pediatric allogeneic hematopoietic stem cell transplantation: a single-center experience

**DOI:** 10.1007/s44313-026-00133-6

**Published:** 2026-04-02

**Authors:** Pai-Lin Tsai, Meng-Yao Lu, Hsiu-Hao Chang, Yung-Li Yang, Chia-Jui Du, Chang-Hsueh Wu, Shiann-Tarng Jou, Shu-Wei Chou

**Affiliations:** 1 Department of Pediatrics, National Taiwan University Hospital, National Taiwan University College of Medicine, Taipei, Taiwan; 2 Department of Pediatrics, National Taiwan University Hospital, Hsin-Chu Branch, Hsin-Chu, Taiwan; 3 Department of Laboratory Medicine, National Taiwan University Hospital, National Taiwan University College of Medicine, Taipei, Taiwan; 4 Department of Laboratory Medicine, College of Medicine, National Taiwan University, Taipei, Taiwan; 5 Department of Laboratory Medicine and Medical Service, National Taiwan University Cancer Center, Taipei, Taiwan; 6 Department of Integrated Diagnostics & Therapeutics, National Taiwan University Hospital, National Taiwan University College of Medicine, Taipei, Taiwan; 7 Department of Pharmacy, National Taiwan University Hospital, National Taiwan University College of Medicine, Taipei, Taiwan

**Keywords:** Cytomegalovirus, Transplantation, Pediatrics

## Abstract

**Purpose:**

Letermovir prophylaxis results in a significantly lower risk of clinically significant cytomegalovirus (CMV) infection in adult hematopoietic stem cell transplant recipients. However, studies supporting its efficacy in pediatric patients are limited.

**Methods:**

We retrospectively reviewed the outcomes of ten patients who underwent allogeneic hematopoietic stem cell transplantation and received letermovir prophylaxis between November 2021 and October 2024 at our institution.

**Results:**

Among the ten patients, six donors were CMV-seropositive, whereas nine recipients were CMV-seropositive. Six patients (60%) underwent transplantation from mismatched unrelated donors, and two (20%) from haploidentical donors. Five patients (50%) developed acute graft-versus-host disease (GvHD) > grade 2.

Ten patients received letermovir prophylaxis for a median of 101.5 days (range, 26–279). The median daily letermovir dose was 4.6 mg/kg (range, 3.5–11.1 mg/kg), with concomitant use of cyclosporin in nine patients. Eight patients (80%) received letermovir as primary prophylaxis, and two (20%) received it as secondary prophylaxis. None of the patients treated with letermovir as primary prophylaxis had CMV reactivation even after discontinuation (median follow-up, 154 days after transplantation, range 37–850). Both patients who received letermovir as secondary prophylaxis showed CMV reactivation. None of the patients developed CMV infection. No significant adverse effects resulting from the letermovir treatment were observed.

**Conclusion:**

Our data support the feasibility of letermovir prophylaxis in pediatric patients. The optimal dose of letermovir in pediatric patients has not been established. Letermovir, used as secondary prophylaxis in pediatric patients, was well tolerated.

**Supplementary Information:**

The online version contains supplementary material available at 10.1007/s44313-026-00133-6.

## Introduction

Cytomegalovirus (CMV) is one of the most common infectious pathogens after allogeneic hematopoietic stem cell transplantation (allo-HSCT) [1]. In a ten-year retrospective study in Taiwan, the cumulative probabilities of CMV infection and CMV disease on day 100 among allo-HSCT recipients were 53.7% and 6.1%, respectively [2]. CMV reactivation is associated with increased non-relapse mortality, resulting in decreased disease-free survival and overall survival following allo-HSCT, and is thus regarded as a severe complication [2–4]. Risk factors for CMV reactivation include CMV-seropositive donor, a mismatched or unrelated donor, myeloablative conditioning, total body irradiation, anti-thymocyte globulin, mycophenolate mofetil, and acute graft-versus-host disease (GvHD) [2, 5]. Thus, CMV prophylaxis is important for allo-HSCT recipients with these risks. Letermovir is an antiviral agent that inhibits the CMV DNA terminase complex, and its prophylactic use has been shown to result in a significantly lower risk of clinically significant CMV infection in adult hematopoietic stem cell transplant recipients, with few mainly low grade adverse events [6]. However, studies supporting its efficacy in pediatric patients are limited.

This study aimed to evaluate the efficacy and safety of letermovir for CMV prophylaxis through a retrospective analysis of pediatric patients who underwent allo-HSCT.

## Methods

We enrolled patients aged < 18 years who underwent allo-HSCT between November 2021 and October 2024 at the National Taiwan University Children’s Hospital. After informed consent was obtained from the parents, letermovir was administered as primary or secondary prophylaxis. Indications for primary prophylaxis included CMV-seropositive donors or recipients, human leukocyte antigen disparity, and unrelated donors. Secondary prophylaxis was defined as letermovir use to prevent recurrence in patients who were successfully treated for clinically significant CMV infection. The baseline characteristics of the patients were reviewed. We analyzed patient outcomes, including CMV reactivation, CMV disease, death, and serious adverse events related to letermovir use.

All patients were routinely screened for CMV viral load once a week after transplantation until discharge, and then weekly or biweekly after discharge, according to the patient’s serostatus. CMV reactivation was defined as CMV viremia of > 150 copies/ml (136.5 IU/ml), and pre-emptive treatment is considered [6]. CMV disease is defined as a CMV infection with attributable symptoms or signs, manifested as either a viral syndrome or tissue-invasive disease. Pre-emptive treatment was initiated either when the serum CMV viral load reached 500 copies/ml or when CMV disease occurred, with individual clinical manifestations considered by the treating physicians. Ganciclovir was used as a first-line antiviral agent. The CMV viral load was monitored biweekly during pre-emptive treatment.

Categorical variables were reported as counts and percentages. This study was conducted in accordance with the principles of the Declaration of Helsinki and approved by the Institutional Review Board (No.202512114RINC) of the National Taiwan University Hospital, Taipei, Taiwan. No funding or other support was received for this study.

## Results

### Patients

Ten patients were enrolled in this study and their baseline characteristics are shown in Table 1. The median age was 10.5 years (range 1–18 years), and six patients (60%) were male. Six donors (60%) and nine recipients (90%) were CMV-seropositive. Seven patients had underlying malignancies, including acute lymphoblastic leukemia (ALL), acute myeloid leukemia (AML), and non-Hodgkin lymphoma, whereas three had other diseases. Six patients (60%) underwent transplantation from mismatched unrelated donors, one (10%) from matched unrelated donors, two (20%) from haploidentical donors, and one (10%) from a matched sibling donor. Seven (70%) patients received a myeloablative conditioning regimen. All the patients received anti-thymocyte globulin serotherapy. For GvHD prophylaxis, all patients received methotrexate; nine (90%) received cyclosporine, and one (10%) received tacrolimus and abatacept instead because of previous hypertension and stroke episodes. Five patients (50%) developed acute GvHD greater than grade 2. Six patients (60%) received corticosteroids: four for acute GvHD treatment, one for idiopathic pulmonary syndrome, and one for a post-transplant lymphoproliferative disorder. Among the ten patients, eight (80%) received letermovir as primary prophylaxis, and two (20%) as secondary prophylaxis. The baseline characteristics of the patients are listed in Table 1 and further details are provided in Supplementary Table 1.

**Table 1:** Characteristics at baseline of all patients (*N* = 10)

Age—yr	
Median	10.5
Range	1–18
Male sex—no. (%)	6 (60)
CMV-seropositive donors—no. (%)	6 (60)
CMV-seropositive recipients—no. (%)	9 (90)
Primary reason for hematopoietic-cell transplantation—no. (%)	
Acute lymphoblastic leukemia	3 (30)
Acute myeloid leukemia	3 (30)
Non-Hodgkin lymphoma	1 (10)
Other diseases	3^a^ (30)
HLA matching and donor type—no. (%)	
Matched unrelated donor	1 (10)
Matched related donor	1 (10)
Mismatched unrelated donor	6 (60)
Haploidentical related donor	2 (20)
Conditioning regimen—no. (%)	
Myeloablative conditioning	7 (70)
Reduced intensity conditioning	3 (30)
Antithymocyte globulin—no. (%)	10(100)
GvHD prophylaxis—no. (%)	
Methotrexate	10 (100)
Cyclosporine	9 (90)
Tacrolimus	1 (10)
Abatacept	1 (10)
Acute GvHD of grade ≥ 2—no. (%)	5 (50)
Corticosteroids use—no. (%)	6^b^ (60)

*CMV* cytomegalovirus, *GVHD* graft-versus-host disease

^a^The 3 patients were respectively diagnosed of severe aplastic anemia, severe combined immunodeficiency, and X-linked lymphoproliferative disease

^b^Among the 6 patients, 4 use corticosteroids as treatment for acute GvHD, one for idiopathic pulmonary syndrome, and one for post-transplant lymphoproliferative disorder

### Letermovir administration

The median time to initiation of letermovir in our cohort was the day of stem cell infusion (range 0–30 days after transplantation). The median dosage of letermovir among the nine patients with concomitant cyclosporin use was 4.6 mg/kg/day (range 3.5–8.5 mg/kg/day). The other patient, a 10-year-old girl weight 43 kg who did not receive cyclosporine for GvHD prophylaxis, received letermovir at a dosage of 480 mg daily (11.1 mg/kg/day). The median duration of letermovir use was 101.5 days (range 26–279 days). One patient discontinued letermovir and switched to pre-emptive treatment because of CMV reactivation. Six patients discontinued letermovir after discharge, one patient discontinued letermovir after 100 days of treatment, and two discontinued letermovir because of mortality unrelated to CMV infection.

### Primary prophylaxis

Considering the high risk of CMV reactivation, eight patients received letermovir as primary prophylaxis. None of the patients experienced CMV reactivation either during or after discontinuation of prophylaxis at a median follow-up time of 154 days (range 37–850 days after transplant).

### Secondary prophylaxis

Two patients received letermovir as secondary prophylaxis to prevent recurrence after successful treatment of a previous clinically significant CMV infection. Both patients developed CMV reactivation after 29 and 43 days of letermovir use. One patient continued letermovir for 95 days under close viral load monitoring; the viral load remained no more than 500 copies/ml and later became undetectable. The other patient switched to pre-emptive treatment after 43 days of letermovir use, with ganciclovir followed by valganciclovir for eight months. None of these two patients developed CMV infection.

### Final outcomes

**Fig. 1:**
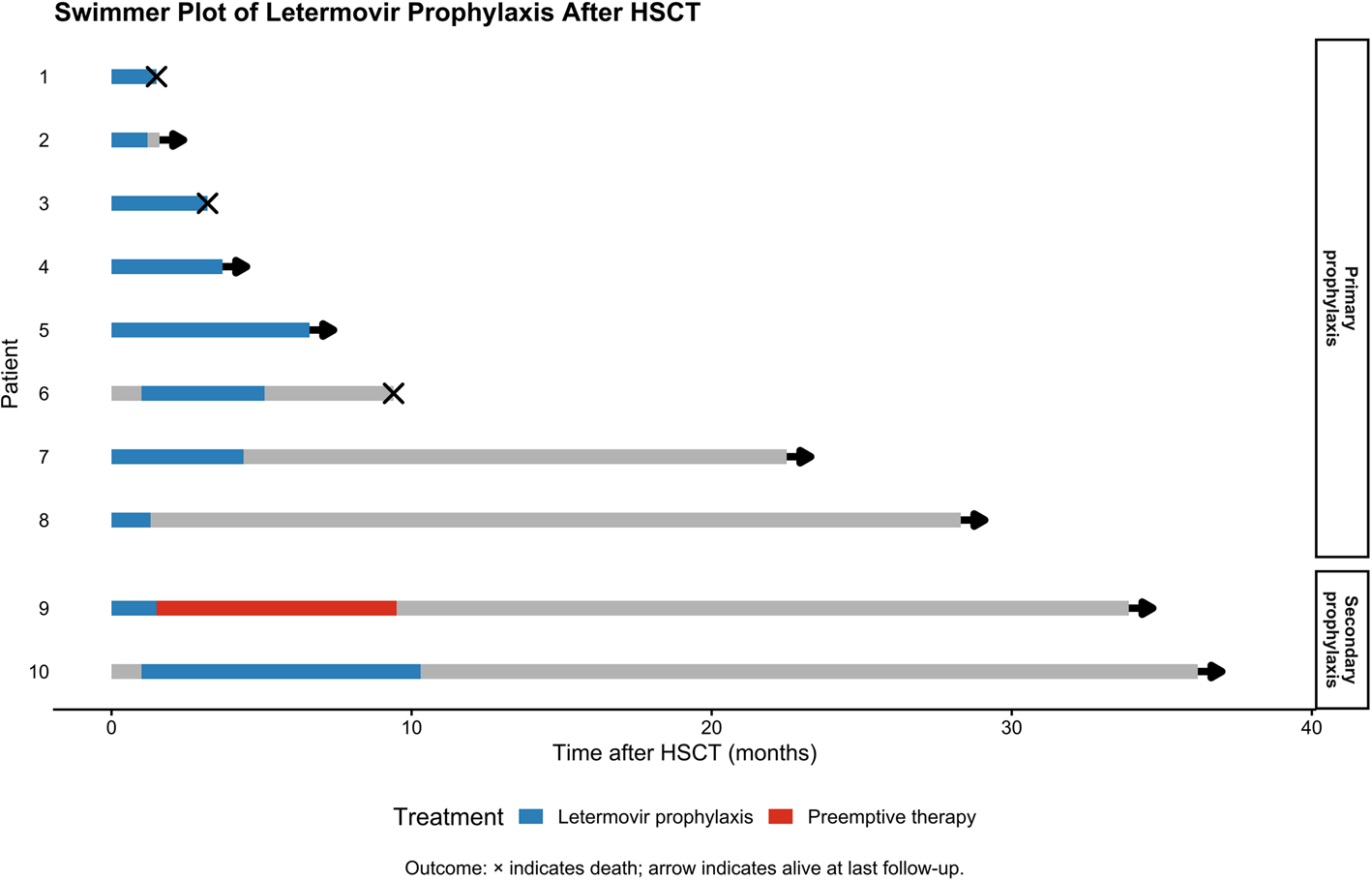
Treatment timelines and clinical outcomes of patients

The median follow-up time of these 10 patients was 240 days (range 37–1086 days after transplant). Three patients died at the end of the follow-up period, two due to bacterial infection and one due to parainfluenza virus pneumonia. None of the patients died of CMV infection. Individual patient treatment timelines and clinical outcomes are illustrated in the swimmer plot in Fig. 1.

### Adverse events

No serious adverse events related to letermovir use were observed in these 10 patients.

## Discussion

In our study, eight patients received letermovir as primary prophylaxis, and there was no CMV reactivation either during prophylaxis or after discontinuation. However, in the two patients who received letermovir as secondary prophylaxis, the CMV reactivation rate was 100%.

In 2020, Styczyński described the use of letermovir for primary prophylaxis of CMV infection in two pediatric cases after allo-HSCT; the two children received 4.1 mg/kg/day and 4.4 mg/kg/day, respectively, with concomitant use of cyclosporine, and the results showed efficacy and safety without serious adverse events [7]. In another retrospective cohort of nine patients aged 4–19 years who underwent allogeneic HCT in the Mayo Clinic Pediatric Bone Marrow Transplant Program, patients whose body weight was less than 30 kg were administered 240 mg/day, and those weighing more than 30 kg were administered 480 mg/day; the median dose for the entire cohort was 10.4 mg/kg/day, all without concomitant cyclosporine [8]. Galaverna reported the youngest patient, an infant aged 8months and weighing 6 kg, who received an 80 mg daily dose [9]. There was no discontinuation due to severe toxicity. On August 30, 2024, letermovir was approved for its expanded indication in pediatric HSCT recipients at high risk of CMV infection. The recommended dose for patients 6 months to less than 12 years of age or 12 years of age and older and weighing less than 30 kg is based on weight, whereas those older than 12 years of age are given 480 mg once daily, either orally or intravenously. In our cohort, the median letermovir dose was 4.6 mg/kg/day (range, 3.5–11.1 mg/kg/day) and appeared effective and well tolerated, consistent with previous studies. This included the youngest patient, a 1-year-old boy weighing 10 kg who received letermovir at 60 mg (6 mg/kg/day) with concomitant cyclosporine.

Marty et al. showed good efficacy of letermovir as a CMV primary prophylaxis in adult HSCT recipients in a phase 3 randomized study [6]. Several cohort studies have also confirmed the efficacy of letermovir, with CMV reactivation rates between 0 and 14% and CMV disease rates between 0% and 0.3% (Table 2). In our study, there was no CMV reactivation or CMV disease in any of the patients receiving letermovir as primary prophylaxis, either during prophylactic treatment or after discontinuation. This result is compatible with data from both the adult and pediatric/adolescent studies [6, 8–11], which are summarized in Table 2.

**Table 2:** Efficacy and safety of letermovir prophylaxis

	Study	CMV Reactivation	CMV Disease	All-cause Mortality	CMV-related Mortality	AE Related to Letermovir
Primary prophylaxis	Marty et al., 2017 [6]	25/325(7.7%)	1/325(0.3%)	20.9%	-	-
	Kuhn et al., 2022 [8]	1/7(14%)	0/7(0%)	0/7(0%)	0/7(0%)	2/9(22%)
	Galaverna et al., 2024 [9]	3/39(7.7%)	0/39(0%)	4/39(10.3%)	0/39(0%)	-
	Cheng et al., 2022 [10]	0/4(0%)	0/4(0%)	0/4 (0%)	0/4 (0%)	4/4(100%)
	NTUCH, 2021–2024	0/8(0%)	0/8(0%)	3/8 (37.5%)	0/8 (0%)	0/8 (0%)
Secondary prophylaxis	Kuhn et al., 2022 [8]	0/2(0%)	0/2(0%)	-	0/2(0%)	2/9(22%)
	Galaverna et al., 2024 [9]	0/26(0%)	1/26(3.8%)	4/26(15.3%)	0/26(0%)	-
	NTUCH, 2021–2024	2/2(100%)	0/2(0%)	0/2(0%)	0/2(0%)	0/2(0%)

*NTUCH* National Taiwan University Children's Hospital

Retrospective adult studies have demonstrated the role of letermovir as a safe secondary prophylaxis, but its efficacy was variable [12, 13]. In the French compassionate program, among 80 HSCT recipients receiving letermovir secondary prophylaxis, four developed breakthrough CMV infections, three of which were CMV diseases [13]. In the Mayo Clinic Pediatric Bone Marrow Transplant Program, letermovir was administered as secondary prophylaxis in two patients after successful treatment of prior CMV viremia/disease [8]. Galaverna et al. presented a retrospective cohort of 26 patients who received letermovir as secondary prophylaxis after HSCT; one patient experienced breakthrough CMV disease during prophylaxis and therefore shifted to pre-emptive treatment, and six developed asymptomatic CMV reactivation/infection after letermovir interruption [9]. In our experience, the CMV reactivation rate in patients receiving letermovir as a secondary prophylaxis was 100% (Table 2). The letermovir dose used in these patients was similar to that reported in previous cohort studies. The long-term use of immunosuppressants may explain these results.

In studies of letermovir for CMV prophylaxis in adult HSCT groups, most adverse events during treatment were gastrointestinal disorders, including diarrhea, nausea, and vomiting, which were only modestly higher in the letermovir group than in the placebo group, and the safety profile of letermovir was also similar to that of placebo [6, 14]. In a Taiwanese pediatric cohort, the rate of adverse events, including gastrointestinal disorders, was not higher in the letermovir group than in the control group [10]. No serious letermovir-related adverse events were recorded.

Because the incidence of clinically significant cytomegalovirus infection increased to as much as 12% between 100 and 200 days after discontinuation of letermovir at day 100, extending letermovir prophylaxis to 200 days after HSCT has been shown to reduce the incidence of late clinically significant cytomegalovirus infection [15]. The median duration in our cohort was 101.5 days (range, 26–279), and five patients received letermovir for over 100 days. Among the ten patients, treatment was discontinued in two because of death, and one shifted to pre-emptive treatment. No CMV reactivation was observed in the other seven patients after the discontinuation of letermovir. This can be explained by the lack of prolonged immunosuppressant use. Therefore, longer-term follow-up is required.

This study had several limitations. This was a single-center retrospective study with a small sample size. The results are significantly affected by individual cases, and conclusions regarding efficacy should therefore be interpreted as preliminary and hypothesis-generating. Population heterogeneity, including underlying diseases, donor types, and conditioning regimens, also limits generalizability. Furthermore, there is no consensus on the optimal duration of letermovir use. Further studies with larger sample sizes and long-term follow-up are necessary.

## Conclusion

Our data support the feasibility of letermovir prophylaxis in the pediatric population and suggest its potential effectiveness in reducing clinically significant CMV reactivation and disease. Moreover, letermovir administration as a secondary prophylaxis in pediatric HSCT patients is safe and well tolerated.

## Supplementary Information

Supplementary Material 1. Supplementary Table S1. Detailed patient characteristics.

## Data Availability

The datasets generated during and/or analyzed during the current study are available from the corresponding author on reasonable request.

## Abbreviations

CMV: Cytomegalovirus
TBI: Total body irradiation
ATG: Anti-thymocyte globulin
GvHD: Graft-versus-host disease
HSCT: Hematopoietic stem cell transplantation
HLA: Human leukocyte antigen
ALL: Acute lymphoblastic leukemia
AML: Acute myeloid leukemia
EBV: Epstein-Barr virus
