# Supplementary material for: Letermovir for cytomegalovirus prophylaxis in pediatric allogeneic hematopoietic stem cell transplantation: a single-center experience
Source: Blood Res. 2026 Apr 2;61(1):13. doi: 10.1007/s44313-026-00133-6 (PMC13047026; doi:10.1007/s44313-026-00133-6)
Supplement: Supplementary file 1 — Supplementary Material 1. Supplementary Table S1. Detailed patient characteristics. [file 44313_2026_133_MOESM1_ESM.docx]

**Supplementary Table S1. Detailed patient characteristics**

| **Case** | **Age**  **(year)** | **Underlying disease** | **Donor source** | **Serotherapy** | **CMV status** | **Grade 2- 4 aGvHD** | **Steroid use** | **LTV initiation** | **LTV duration (days)** | **LTV dose (mg)** | **CMV reactivation** | **CMV disease** | **Final Outcomes** |
| --- | --- | --- | --- | --- | --- | --- | --- | --- | --- | --- | --- | --- | --- |
| 1 | 2 | EBV+ T cell lymphoma | Haplo | ATG | D-/R+ | (-) | (+) | D0 | 38 | 60 (5mg/kg) | (-) | (-) | Died  *Parainfluenza pneumonia |
| 2 | 10 | SAA | MMUD | ATG | D-/R+ | (-) | (-) | D+1 | 26 | 480 (11.1mg/kg) | (-) | (-) | Alive |
| 3 | 10 | T-ALL | Haplo | ATG | D-/R+ | (+) | (+) | D0 | 98 | 240 (8.5mg/kg) | (-) | (-) | Died  *Bacterial infection |
| 4 | 11 | AML | MMUD | ATG | D+/R- | (+) | (-) | D0 | 105 | 120 (3.6mg/kg) | (-) | (-) | Alive |
| 5 | 11 | T-ALL | MMUD | ATG | D-/R+ | (+) | (+) | D0 | 190 | 240 (5.3mg/kg) | (-) | (-) | Alive |
| 6 | 16 | AML | MMUD | ATG | D+/R+ | (-) | (+) | D+29 | 121 | 240 (3.5mg/kg) | (-) | (-) | Died  *Bacterial infection |
| 7 | 1 | AML | MUD | ATG | D+/R+ | (-) | (-) | D0 | 133 | 60 (6mg/kg) | (-) | (-) | Alive |
| 8 | 18 | B-ALL | MMUD | ATG | D+/R+ | (-) | (-) | D0 | 39 | 240 (4.1mg/kg) | (-) | (-) | Alive |
| 9 | 2 | SCID | MMUD | ATG | D+/R+ | (+) | (+) | D0 | 50 | 50 (4mg/kg) | (+) | (-) | Alive |
| 10 | 16 | XLP | MSD | ATG | D+/R+ | (+) | (+) | D+30 | 279 | 240 (3.7mg/kg) | (+) | (-) | Alive |

aGvHD: acute graft-versus-host disease; ALL: acute lymphoblastic leukemia; AML: acute myeloid leukemia; ATG: anti-thymocyte globulin; CMV: cytomegalovirus; D+: donor seropositive; D-: donor seronegative; Haplo: haploidentical; LTV: letermovir; MMUD: mismatched unrelated donor; MSD: matched sibling donor; MUD: matched unrelated donor; R+: recipient seropositive; R-: recipient seronegative; SAA: severe aplastic anemia; SCID: severe combined immunodeficiency; XLP: X-linked lymphoproliferative disease
